# Pre-test ^68^Ga-PSMA-ligand PET/CT positivity in early biochemical recurrent prostate cancer after radical prostatectomy—validation of a prediction model

**DOI:** 10.1186/s13550-020-0595-5

**Published:** 2020-02-03

**Authors:** Pia Kraft, Tobias Maurer, Andrei Gafita, Markus Krönke, Bernhard Haller, Wolfgang A. Weber, Matthias Eiber, Isabel Rauscher

**Affiliations:** 1 School of Medicine, Klinikum rechts der Isar, Department of Nuclear Medicine, Technical University of Munich, Ismaninger Str. 22, 81675 Munich, Germany; 2 School of Medicine, Klinikum rechts der Isar, Department of Urology, Technical University of Munich, Ismaninger Str. 22, 81675 Munich, Germany; 3 School of Medicine, Klinikum rechts der Isar, Institute of Medical Informatics, Statistics and Epidemiology, Technical University of Munich, Ismaninger Str. 22, 81675 Munich, Germany; 4 Department of Urology, Martini-Klinik Prostate Cancer Center, University Hospital Hamburg-Eppendorf, Martini-Str. 52, 20246 Hamburg, Germany

**Keywords:** Biochemical recurrence, Prostate cancer, PSMA, External validation, Prediction model

## Abstract

**Objectives:**

The aim of this study was the validation of a recently established comprehensive and compact prediction model for ^68^Ga-PSMA-11-ligand positron-emission tomography (PET) positivity with an independent subsequent patient series.

**Methods:**

A total of 292 consecutive patients with early biochemical recurrence after radical prostatectomy and PSA values between 0.2 and 1 ng/ml who underwent ^68^Ga-PSMA-11-ligand PET/computed tomography (CT) between January 2016 and June 2017 were retrospectively included. The cohort was divided into a very low PSA value (0.2–0.5 ng/ml, *n* = 151) and a low PSA value (> 0.5–1 ng/ml, *n* = 141) subgroup. First, pre-test positivity probabilities for each patient were calculated according to the previously published comprehensive prediction model using all clinical variables (PSA value, ISUP grade group, T- and N-stage, patient under androgen deprivation therapy (ADT), previous radiation therapy) and the compact model using just the most predictive factors PSA value, ADT, and grade group. Then, all ^68^Ga-PSMA-11-ligand PET/CTs were analysed by one experienced nuclear medicine physician, and the results were correlated to the calculated pre-test probabilities.

**Results:**

In the very low PSA value subgroup, mean pre-test probability for positive findings in ^68^Ga-PSMA-11-ligand PET/CT was 57% (95% CI 55–60%) according to the compact model and 59% (95% CI 56–61%) according to the comprehensive model. In the low PSA value subgroup, mean pre-test probability was 72% (95% CI 70–74%) in the compact model and 74% (95% CI 72–76%) in the comprehensive model. After image analysis, 59% (89/151) of the patients in the very low PSA value subgroup revealed positive imaging findings. Seventy-nine percent (112/141) of the patients in the low PSA value subgroup presented with positive findings in the ^68^Ga-PSMA-11-ligand PET/CT. The accuracy (AUC) of the prediction models was 0.71 (95% CI 0.65–0.78) for the compact model and 0.74 (95% CI 0.68–0.80) for the comprehensive model.

**Conclusion:**

External validation of the recently proposed prediction models showed a high concordance of the calculated pre-test probabilities and actual ^68^Ga-PSMA-11-ligand PET/CT findings in the validation cohort confirming the prediction models’ ability to determine the presence of a positive lesion at ^68^Ga-PSMA-11-ligand PET. However, the predictive accuracy of the nomogram itself is suboptimal and should be used with caution. Furthermore, the model’s generalizability may be hampered due to the study design (in-house validation). Nevertheless, given the limited health resources and the costs of hybrid imaging techniques, prediction models might be a benefit in patient selection.

## Introduction

Prostate cancer (PC) is the second most common cancer in men worldwide [1]. After primary radical prostatectomy (RP), the recurrence rate ranges between 7 and 50% within 5 years after surgery [2, 3]. In these cases, early detection and localization of recurrent disease is crucial for further disease management [4, 5]. Conventional imaging such as computed tomography (CT) or magnetic resonance imaging as well as choline-based PET often fails to localize biochemical recurrence [4, 6–8]. Since its first introduction in 2014, ^68^Ga-PSMA-11-ligand positron emission tomography/computed tomography (PET/CT) has been adopted rapidly around the world given its high sensitivity and specificity for the detection and localization PC lesions. The most accepted clinical indication for PSMA-ligand PET/CT is biochemically recurrent PC as various studies indicate superior detection efficacy in comparison to conventional imaging and choline-based PET, even at low PSA levels [9]. This is also reflected by the recent introduction into the EAU guidelines [4].

In uro-oncology, prediction nomograms are designed to help patients and their physicians to assess risk based on specific characteristics and predict the likely outcomes of treatment. In PC, the most established nomograms are the Partin tables or the Kattan nomograms predicting, for example, the extent of the cancer and long-term results following RP already prior surgery or the probability of remaining cancer recurrence-free after surgery [10, 11]. Recently, both a compact and a full comprehensive version of a prediction model were introduced providing individualized predictions for positive ^68^Ga-PSMA-11-ligand PET/CT scan in patients with very early biochemical recurrence (PSA 0.2–1 ng/ml). These can assist urologists in discussions with their patients on the use of ^68^Ga-PSMA-11-ligand PET/CT in specific clinical settings [12]. The compact model combines the three most predictive clinical variables (PSA level, androgen deprivation therapy (ADT), ISUP Grade Group (old Gleason score)) which is in line with several recent studies supporting the role of PSA, ADT, and Gleason score as independent predictive factors for PSMA-ligand PET positivity [12–14]. The comprehensive model adds the variables primary T- and N-stage, prior radiation therapy, and time interval from RP to PSMA-ligand PET/CT examination for calculation of PSMA-ligand PET positivity.

In order to further establish the previously proposed prediction models showing their applicability in different patient populations, we aimed to validate the prediction models estimating pre-test ^68^Ga-PSMA-11-ligand PET/CT positivity in early biochemical recurrent PC patients after RP in a second, independent patient population.

## Material and methods

### Patient population

**Fig. 1:**
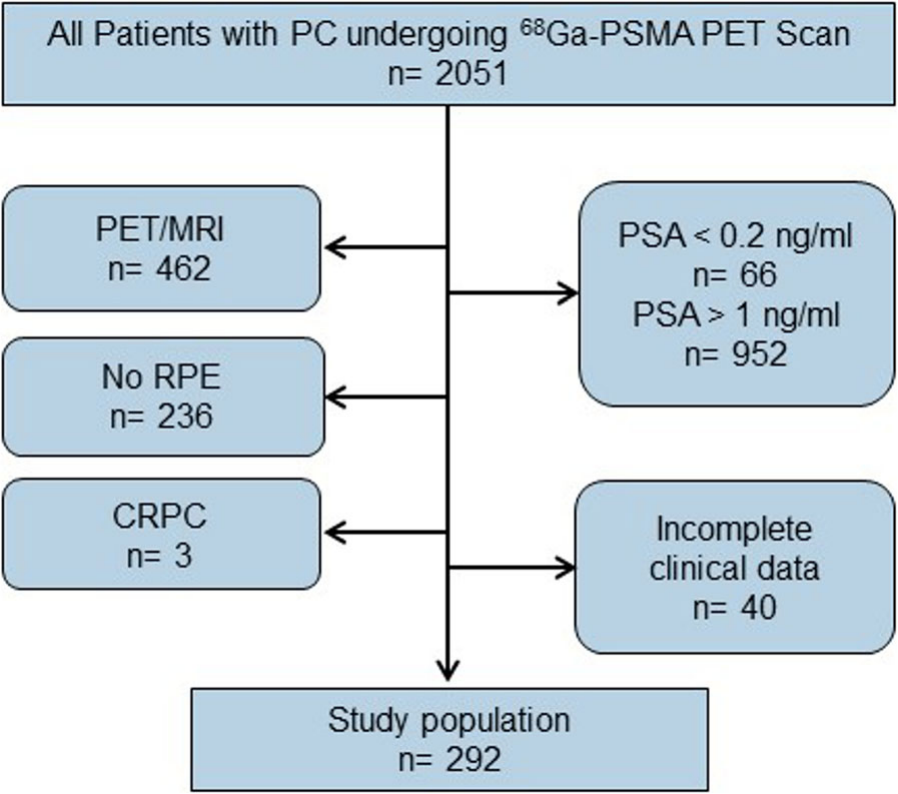
Patient selection flow chart. PC, prostate cancer; RP, radical prostatectomy; CRPC, castration resistant prostate cancer

We extracted 2051 consecutive patients undergoing ^68^Ga-PSMA-ligand PET imaging from the institution’s database (January 2016 to June 2017). Only patients who were examined by PET/CT and showed PSA values from 0.2 up to 1 ng/ml at time of ^68^Ga-PSMA-11-ligand PET after primary treatment with RP were included in this study. Details on patient selection are presented in Fig. 1. If several ^68^Ga-PSMA-11-ligand PET/CT examinations within one patient were available, only the first examination was included. All patients signed a written informed consent form for the purpose of anonymized evaluation and publication of their data. The study was approved by the Ethics Committee of the Technical University Munich (permit 5665/13).

### ^68^Ga-PSMA-11-ligand PET/CT imaging

Images were obtained with the ^68^Ga-labelled HBED-CC that was synthesized as described previously [15]. ^68^Ga-PSMA-11-ligand was applied to patients via an intravenous bolus (mean 1.76 MBq/kg body weight, IQR 1.18–1.94 MBq/kg; mean 131.5 MBq, IQR 102.3–160.6 MBq). PET acquisition was started at a mean time of 54.1 ± 11.3 min after tracer injection (range 40–181 min; for two patients, compensatory increase in time per bed position due to late imaging acquisition). All patients underwent ^68^Ga-PSMA-11-ligand PET/CT on a Biograph mCT scanner (Siemens Medical Solutions, Erlangen, Germany). First, a diagnostic CT scan was performed in the portal venous phase 80 s after intravenous injection of contrast agent (Imeron 300) followed by the PET scan. All PET scans were acquired in 3D mode with an acquisition time of 3–4 min per bed position. Emission data were corrected for randoms, dead time, scatter, and attenuation and were reconstructed iteratively by an ordered-subsets expectation maximization algorithm (four iterations, eight subsets) followed by a postreconstruction smoothing Gaussian filter (5-mm full width at one-half maximum).

### Data analysis

First, a pre-test positivity probability score (percentage with 95% confidence intervals) was calculated for each patient using the formula of the compact and the comprehensive prediction model described in Rauscher et al. [12]. In the second step, all ^68^Ga-PSMA-11-ligand PET/CTs were evaluated by one experienced nuclear medicine physician (IR) blinded to the results of the prediction model and all clinical data of the patient [16, 17]. All lesions suspicious for recurrent PC were noted and grouped into the following: (a) local recurrence, (b) lymph node metastases (pelvic/retroperitoneal vs. supradiaphragmatic location), (c) bone metastases, and (d) other visceral metastases (e.g. lung, liver, spleen).

### Statistical analysis

All statistical analyses were performed with the SPSS software. For analysis, the patient cohort was divided into two subgroups: patients with very low PSA values (0.2–0.5 ng/ml) and patients with low PSA values (> 0.5–1 ng/ml). Pre-test probabilities were calculated as previously described and correlated with the results from subsequent ^68^Ga-PSMA-11-ligand PET/CT analysis for all patients in each PSA subgroup [12]. Logistic regression models were fitted to the data to estimate odds ratios for *t* variables. Receiver operating characteristics (ROC) analysis was conducted to compare associations of binary variables to ^68^Ga-PSMA-11-ligand PET positivity and the discriminatory ability of the prediction model. To validate the predicted and actual probabilities, calibration plots were calculated. All statistical tests were performed two-sided, and a significance level of α = 5% was used.

## Results

In total, 292 patients were included in this study. Patient characteristics are summarized in Table 1. One hundred fifty-one patients presented with very low PSA values (0.2–0.5 ng/ml) and 141 patients presented with low PSA values (> 0.5–1 ng/ml), respectively.

**Table 1:** Patients’ clinical and pathologic characteristics

Characteristics	*n* = 292	
Age at PET/CT, median (years)		70 (IQR 65–74)
Primary Gleason score at RP	≤ 7	204 (69.9%)
	≥ 8	88 (30.1%)
Pathologic primary tumour stage (pT)	≤ pT2c	140 (47.9%)
	≥ pT3a	152 (52.1%)
Pathologic regional lymph node stage (pN)	pN0	230 (78.8%)
	pN1	62 (21.2%)
Additional treatment after RP^1^		142 (48.6%)
Radiation therapy		107 (36.6%)
ADT (within the last 6 months)		35 (12.0%)
PSA value prior to PET/CT, median (ng/ml)		0.50 (IQR 0.35–0.70)
0.2–0.5 ng/ml (very low)	*n* = 151	0.35 (IQR 0.27–0.43)
> 0.5–1.0 ng/ml (low)	*n* = 141	0.71 (IQR 0.60–0.90)

*ADT* androgen deprivation therapy, *RP* radical prostatectomy

^1^Multiple secondary treatments within a patient possible

### Calculated pre-test probabilities

In the very low PSA value subgroup (0.2–0.5 ng/ml), mean pre-test probability for positive findings in ^68^Ga-PSMA-11-ligand PET/CT was calculated to be 57% (95% CI 55–60%) according to the compact model and 59% (95% CI 56–61%) according to the comprehensive model. For the low PSA value subgroup (> 0.5–1 ng/ml), the estimated mean pre-test probability was 72% (95% CI 70–74%) in the compact model and 74% (95% CI 72–76%) in the comprehensive model.

### Lesion detection and localization

In total, ^68^Ga-PSMA-11-ligand PET/CT was positive in 68.8% (201/292) of all patients in the validation cohort, respectively. In the very low PSA value subgroup (0.2–0.5 ng/ml), 59% (89/151) of the patients presented with positive imaging findings while 79% (112/141) of the patients in the low PSA value subgroup (> 0.5–1 ng/ml) had a positive ^68^Ga-PSMA-11-ligand PET/CT scan (Table 2). The main site of lesions was pelvic/retroperitoneal lymph nodes, followed by bone and local recurrence. Detailed information on the localization of positive findings on ^68^Ga-PSMA-11-ligand PET according to PSA range is presented in Table 3. Representative examples are shown in Additional file 1: Figure S1.

**Table 2:** Subgroup analysis of pre-test probability and actual positive findings in ^68^Ga-PSMA-11-ligand PET

Patient subgroup	Compact model pre-test probability	Comprehensive model pre-test probability	Positive imaging findings
Entire cohort	67% (95% CI 65–68%)	69% (95% CI 66–71%)	69% (201/292)
Very low PSA (0.2–0.5 ng/ml)	57% (95% CI 55–60%)	59% (95% CI 56–61%)	59% (89/151)
Low PSA (> 0.5–1 ng/ml)	72% (95% CI 70–74%)	74% (95% CI 72–76%)	79% (112/141)

**Table 3:** Localization of positive findings on ^68^Ga-PSMA-11-ligand PET according to PSA range

PSA range	0.2–0.5 ng/ml (very low)		> 0.5–1.0 ng/ml (low)		*p* value
	No.	%	No.	%	
Total no. of patients with positive findings	89/151	58.9	112/141	79.4	0.0003*
Localization of positive findings on ^68^Ga-PSMA-11-ligand PET					
Local	24/151	15.9	38/141	27.0	0.0297*
LN pelvic/retroperitoneal	58/151	38.4	73/141	51.8	0.0290*
LN supradiaphragmal	7/151	4.6	7/141	5.0	0.9091
Bone	30/151	19.9	28/141	19.9	0.8834
Visceral	2/151	1.3	4/141	2.8	0.6215

*Significant difference *p* ≤ 0.05

### Accuracy of the prediction models

**Fig. 2:**
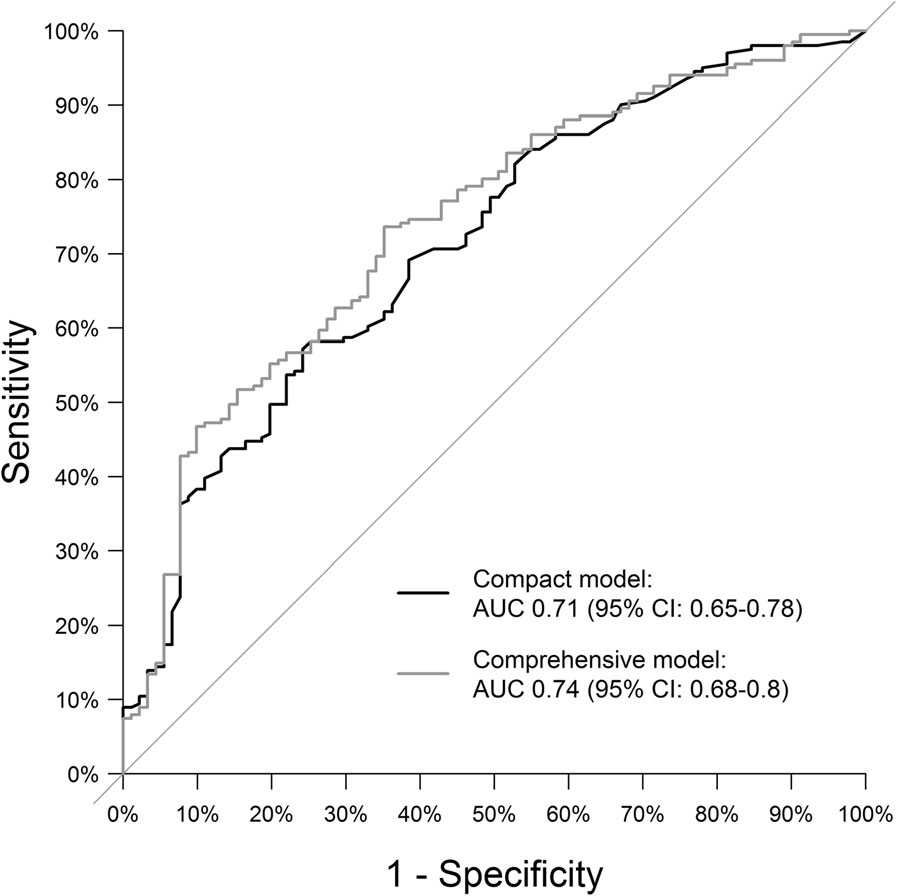
ROC curves of the prediction models. The black line corresponds to the compact model, and the grey line to the comprehensive model (AUC, area under the curve; CI, confidence interval; *p*-values comparison to AUC = 0.5)

In our study, the accuracy (area under the ROC curve (AUC)) of the prediction models was 0.71 (95% confidence interval (CI) 0.65–0.78) for the compact model and 0.74 (95% confidence interval 0.68–0.80) for the comprehensive model (Fig. 2). This indicates a moderate to good discriminatory ability. Further, no significant differences were observed compared to the AUCs of our previously published prediction models (*p* values 0.376 and 0.242 in the compact and comprehensive model, respectively) [12].

**Fig. 3:**
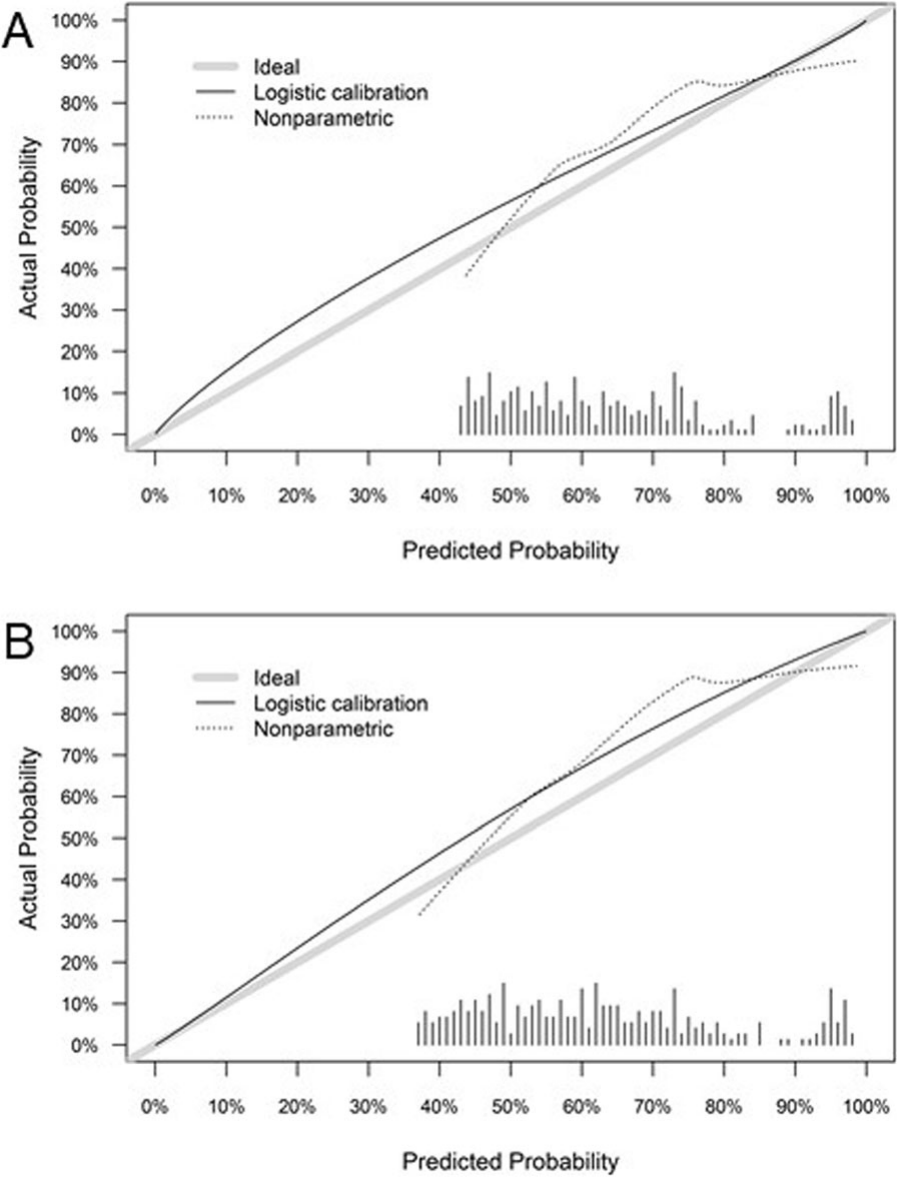
Local regression nonparametric smoothing (calibration) plot of the compact (**a**) and comprehensive (**b**) prediction model showing the relationship between nomogram predictions and observed frequency of positive ^68^Ga-PSMA-11-ligand PET/CT examinations in the validation cohort. Perfect or ideal predictions correspond to the 45° line (grey line). Points situated above the 45° line correspond to overprediction in the validation cohort in comparison to the prediction model, whereas points estimated below the 45° line correspond to underestimation in the validation cohort according to the prediction model (dotted black line). Vertical lines indicate the frequency distribution of predicted probabilities

Figure 3 displays the nomogram calibration plot as applied to the validation dataset. The predicted probability of the previously reported prediction model is represented on the *x*-axis, and the actual proportion of ^68^Ga-PSMA-11-ligand PET positivity is represented on the *y*-axis. In both the compact and comprehensive models, a tendency to slightly overestimate the actual positivity rate was observed for most of the patients. Only in patients with a very high or very low predicted positivity rate a slight underestimation of PET positivity was observed.

### Lesion validation

Validation of positive ^68^Ga-PSMA-11-ligand PET/CT findings was available in 137/201 patients (68 %) with at least 1 of the following procedures: (a) targeted radiation therapy with consecutive PSA decline ≤ 0.2 ng/ml (*n* = 56), (b) positive histopathology after salvage lymph node dissection (*n* = 27), and (c) follow-up ^68^Ga-PSMA-11-ligand PET/CT confirming the initial suspicious lesion(s) or disappearance of suspected metastatic sites after local/systemic treatment and corresponding PSA decline (*n* = 17).

## Discussion

Recently, two prediction models to estimate the a priori probability of ^68^Ga-PSMA-ligand PET/CT positivity in patients after RP presenting with biochemical recurrence (PSA level 0.2–1.0 ng/ml) based on a variety of different clinical variables were established. The results of our validation study show a high concordance of the calculated pre-test probabilities and actual ^68^Ga-PSMA-11-ligand PET/CT findings in our recent patient cohort confirming the prediction models’ ability to determine the probability of the presence of a positive lesion at ^68^Ga-PSMA-11-ligand PET (calculated probability: very low PSA 59%/57% and low PSA 74%/72% for the comprehensive/compact model; suspicious lesions observed in ^68^Ga-PSMA-11-ligand PET 59% and 79% for very low and low PSA subgroup, respectively). The predicted pre-test probabilities showed a slightly better congruence for the very low PSA subgroup; however, no statistical difference was observed. As there was no significant difference between the compact and comprehensive prediction model in this validation study, we recommend using the compact model for routine clinical use as several variables included in the original comprehensive model showed no statistical significance at multivariable analysis (e.g. T- and N-stage, radiation therapy after surgery). The calculation of the compact model is easier and faster to apply as less clinical variables are necessary (only PSA level, grade group, and prescription of ADT).

The observed detection rate in our study is slightly higher than that reported in our previously published study (69% (201/292 patients) vs. 65% (176/272 patients) and in comparison to a study of Afshar-Oromieh et al. (60%; 137/227) in patients with a PSA value of 0.2–1 ng/ml [12, 14]. However, it has to be stated that the variance in detection rates is quite high in the literature and the numbers reported in our analysis are within these variations [18]. These variations might be due to the use of different types of PET/CT cameras, acquisition parameters, reconstruction algorithms, and/or the sensitivity/experience of the reader. In order to standardize image acquisition and evaluation and thus improve accuracy, precision, and repeatability of ^68^Ga-PSMA ligand PET/CT examinations, several papers have been published so far including the EANM/SNMMI procedure guidelines on ^68^Ga-PSMA-ligand PET/CT [16, 17, 19, 20]. Nevertheless, further efforts are needed for implementation in routine clinical practice as robust evidence is still lacking.

^68^Ga-PSMA-ligand PET/CT does not only detect but also localize the extent of recurrent disease. The main site of metastatic lesions in our study was pelvic/retroperitoneal lymph nodes, followed by bone and local recurrence which is in line with previous studies [12, 21]. Further, a substantial number of patients presented with supradiaphragmatic lymph node, bone, or visceral metastases, even at PSA values 0.2–0.5 ng/ml (see also Table 3). In patients with a PSA increase up to 0.5 ng/ml, current guidelines recommend salvage radiotherapy. However, approximately 40% of them will not achieve an undetectable PSA value. Thus, ^68^Ga-PSMA-ligand PET/CT might not only modify potentially unsuccessful local salvage radiotherapy but also enable targeted surgical salvage procedures such as PSMA-radioguided surgery [22, 23].

The prediction model itself is not able to specify probabilities below 40% and 30% in the compact and comprehensive model, respectively. Therefore, no justifiable cut-off can be defined in which clinical condition a ^68^Ga-PSMA-11-ligand PET examination is not required. Nevertheless, the prediction model is able to identify patients with recurrent disease in ^68^Ga-PSMA-11-ligand PET/CT with up to 98% certainty (highest score reachable in the prediction model based on clinical variables) who will potentially profit from lesion-targeted treatment. Therefore, the prediction models should rather be understood as a tool for a positive decision towards a PSMA-ligand PET/CT examination. As the results of the AUC were concordant in the patient cohort used for establishing the prediction nomogram and the one used for validation showing no significant difference, a further calibration of the prediction model based on our findings would not bring any added value and was not the aim of our study as well.

Nevertheless, we believe that it is of great interest to establish prediction nomograms being able to identify patients in which the examination will lead to a change in clinical management and might improve oncologic survivals. Thus, in patients at low risk of imaging-detected recurrence, PSMA-ligand PET/CT might not be recommended sparing expensive imaging that would not solve the clinical doubt of tumour recurrence and subsequent management would not have been changed as well. The huge interest in nomograms predicting PSMA-ligand PET positivity is also reflected by the recent introduction of a new prediction nomogram by Ceci et al. [24]. The main difference to the nomogram we validated is that Ceci et al. implemented PSA doubling time and included different clinical settings (first-time biochemical recurrence, biochemical recurrence after salvage therapy, biochemical persistence after radical prostatectomy, and advanced-stage PC before second-line systemic therapies). The predicted accuracy was 82% in their study and thus substantially higher than using the prediction nomograms of Rauscher et al. Further, a 40% probability threshold was identified to be the most accurate cut-off in counselling patients to 68Ga-PSMA-11-PET/CT. However, multicentre external validation of their nomogram is still pending and thus, at present, cannot be suggested in routine.

There are several limitations to our study. First, we examined a subsequent patient cohort for external validation of the prediction model provided from the same institute using the same PET/CT scanner. To be precise, the patient group studied should have been from another site and using a different PET/CT scanner as variation of system performance is a known problem [25]. Therefore, the generalizability of the study may be hampered and multicentre validation in different sites is still mandatory. Nevertheless, we believe the nomogram is applicable on external patient populations as well as the study analyses a well-defined patient cohort meeting strict inclusion criteria. However, image acquisition parameters should be in line with the joint EANM/SNMMI guideline [19]. Further, in our study, ^68^Ga-PSMA-11 was used as PSMA-ligand although recently ^18^F-PSMA-ligands are increasingly used because of various logistical advantages [13]. In future nomograms for ^18^F-PSMA-ligands, it can be anticipated that the low or even no excretion via the urinary tract increases detection probability in metastatic lesions located directly adjacent to the urinary bladder.

Recently, a higher number of lesions with increased PSMA-ligand uptake attributed to benign origin were observed in ^18^F-labelled PSMA-ligand PET in comparison to ^68^Ga-PSMA-11 [26]. However, we do not think that this influences the correct establishment of a prediction nomogram as after adequate reader training, physicians should be well equipped to distinguish likely malignant lesions from those that are most probably benign. However, multivariable regression analysis with subsequent establishment of a prediction nomogram for ^18^F-PSMA-ligand PET still has to show its practicability in the future. In addition, validation of PSMA-ligand positive imaging finding was not available in all patients; however, high specificity of ^68^Ga-PSMA-11-ligand PET has been proven in several studies by now [6, 21].

Above all, precise data proving that PSMA-guided therapy improves the disease-free and overall survival compared to conventional therapy is currently missing. Studies embracing this specific question are absolutely required in the future. At this point, prediction models would be a valuable tool to pre-clinically select patients in which a PSMA PET/CT would be beneficial.

## Conclusion

Our study shows a high concordance of the calculated pre-test probabilities and ^68^Ga-PSMA-11-ligand PET/CT findings confirming the prediction models’ ability to determine the presence of a positive lesion at ^68^Ga-PSMA-11-ligand PET. However, the predictive accuracy of the nomogram itself is suboptimal and should be used with caution. In addition, due to in-house validation, the model’s generalizability may be reduced. Nevertheless, given the limited health resources and the costs of hybrid imaging techniques, prediction models might be a benefit in patient selection.

## Supplementary information

**Additional file 1: Figure S1.** Examples of ^68^Ga-PSMA-11-ligand PET/CT examinations in patients with early recurrent PC after RP. Upper row shows CT datasets, lower row fused PSMA-ligand PET/CT studies. A, D: 58-year old male with biochemical recurrence (PSA 0.92 ng/ml) 8 years after RP (pT2c, pN0, R0, GS 6) with intense, focal PSMA-ligand uptake in the central prostate fossa highly suggestive of local recurrence which was histopathologically proven (positive in HE staining and PSMA immunohistochemistry staining) after salvage surgery. B, E: 70-year old male with biochemical recurrence (PSA 0.21 ng/ml) 2 years after RP (pT3a, pN0, R0, GS 7b) with intense PSMA-ligand uptake in a morphologically unobtrusive lymph node presacral highly suggestive of a single lymph node metastasis. Metastatic involvement of this lymph node was histologically proven (positive in HE staining and PSMA immunohistochemistry staining) after salvage lymphadenectomy. C, F: 67-year old male with biochemical recurrence (PSA 0.35 ng/ml) 5 years after RP (pT3b, pN1, R0, GS 7) with intense accumulation of PSMA tracer in the right pelvic bone highly suggestive of bone metastasis. Follow-up PET/CT after salvage radiation therapy revealed no PSMA-ligand uptake anymore with corresponding PSA decline.

## Data Availability

All data generated or analysed during this study are included in this published article (and its supplementary information files).
