# Supplementary figures and images for: Pre-test 68Ga-PSMA-ligand PET/CT positivity in early biochemical recurrent prostate cancer after radical prostatectomy—validation of a prediction model
Source: EJNMMI Res. 2020 Feb 3;10:6. doi: 10.1186/s13550-020-0595-5 (PMC6997317; doi:10.1186/s13550-020-0595-5)

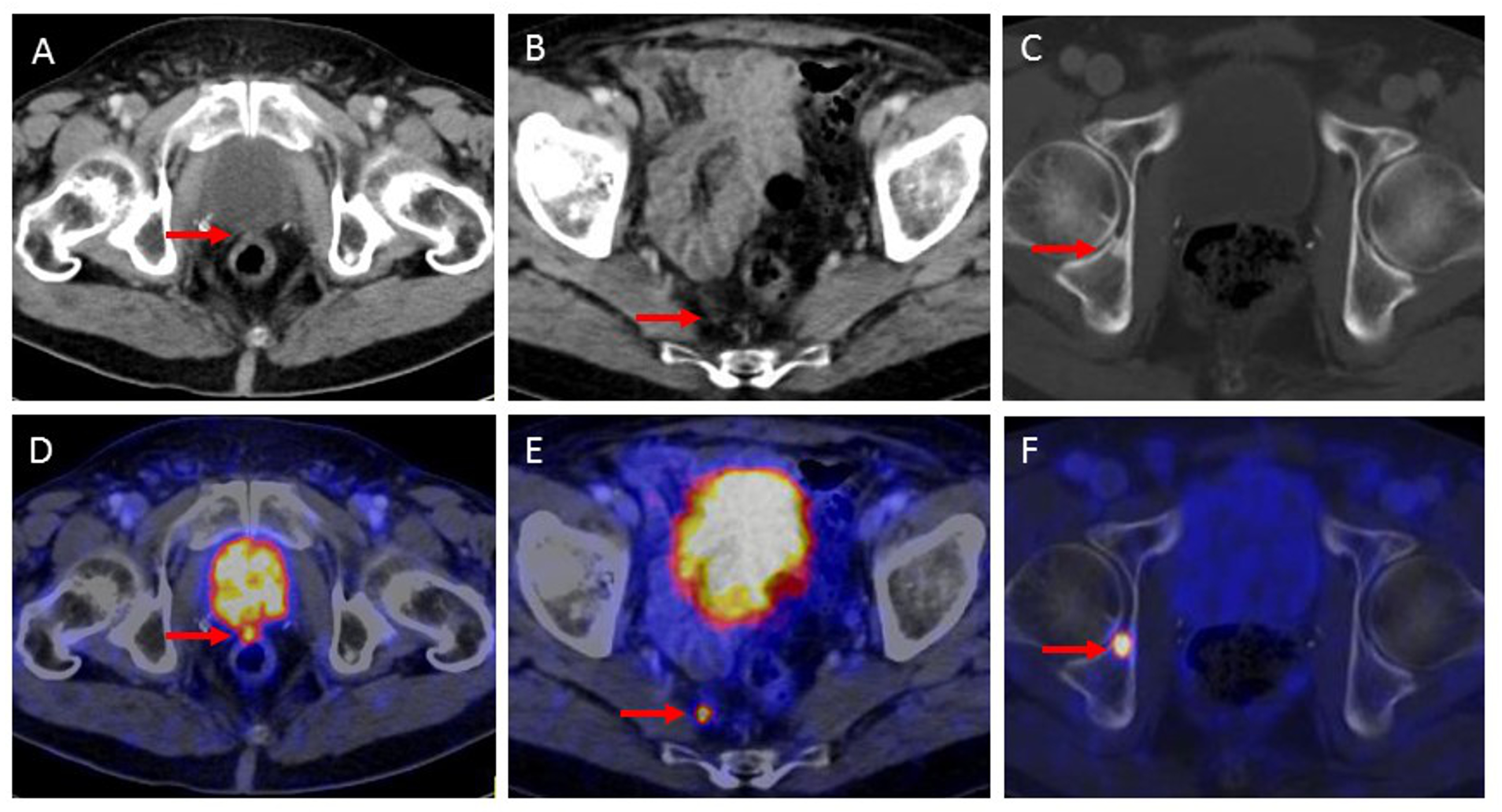

Supplement: Supplementary file 1 — Additional file 1: Figure S1. Examples of 68Ga-PSMA-11-ligand PET/CT examinations in patients with early recurrent PC after RP. Upper row shows CT datasets, lower row fused PSMA-ligand PET/CT studies. A, D: 58-year old male with biochemical recurrence (PSA 0.92 ng/ml) 8 years after RP (pT2c, pN0, R0, GS 6) with intense, focal PSMA-ligand uptake in the central prostate fossa highly suggestive of local recurrence which was histopathologically proven (positive in HE staining and PSMA immunohistochemistry staining) after salvage surgery. B, E: 70-year old male with biochemical recurrence (PSA 0.21 ng/ml) 2 years after RP (pT3a, pN0, R0, GS 7b) with intense PSMA-ligand uptake in a morphologically unobtrusive lymph node presacral highly suggestive of a single lymph node metastasis. Metastatic involvement of this lymph node was histologically proven (positive in HE staining and PSMA immunohistochemistry staining) after salvage lymphadenectomy. C, F: 67-year old male with biochemical recurrence (PSA 0.35 ng/ml) 5 years after RP (pT3b, pN1, R0, GS 7) with intense accumulation of PSMA tracer in the right pelvic bone highly suggestive of bone metastasis. Follow-up PET/CT after salvage radiation therapy revealed no PSMA-ligand uptake anymore with corresponding PSA decline. [file 13550_2020_595_MOESM1_ESM.jpg]
